# Autophagy impairs the sensitivity of Ewing sarcoma cells to PARP inhibitors

**DOI:** 10.1007/s00280-026-04907-8

**Published:** 2026-05-30

**Authors:** Julia Clausen, Daniela Kocher, Hauke M. Schadwinkel, Sabine Becker, Till Milde, James F. Beck, Jürgen Sonnemann

**Affiliations:** 1 Department of Paediatric and Adolescent Medicine, Jena University Hospital, Friedrich Schiller University Jena, Jena, Germany; 2 Research Centre Lobeda, Jena University Hospital, Friedrich Schiller University Jena, Jena, Germany; 3 Comprehensive Cancer Centre Central Germany (CCCG), Jena, Germany; 4 Department of Ophthalmology, UKE Hamburg, Hamburg, Germany; 5 Hopp Children’s Cancer Center Heidelberg (KiTZ), Heidelberg, Germany; 6 Clinical Cooperation Unit Pediatric Oncology, German Cancer Research Center Heidelberg (DKFZ), Heidelberg, Germany

**Keywords:** Ewing sarcoma, PARP, Olaparib, Celiparib, Autophagy, Cyto-ID, Cell sorting

## Abstract

**Purpose:**

PARP inhibitors (PARPi) proved effective in Ewing sarcoma cells in preclinical studies. In clinical evaluation, however, the PARPi olaparib failed to elicit substantial responses, suggesting an unknown mechanism of resistance to PARPi in Ewing sarcoma. Since autophagy has been identified as a PARPi resistance mechanism in other tumours, this study aimed at exploring the impact of autophagy on PARPi effectiveness in Ewing sarcoma cells.

**Methods:**

Effects of the PARPi olaparib and veliparib were assessed by flow cytometry in the Ewing sarcoma cell lines WE-68 and SK-ES-1. Autophagy levels were determined using the autophagosome tracer dye Cyto-ID, and cytotoxic effects were determined by the analysis of cell death and loss of mitochondrial membrane potential. Cyto-ID was used to separate cell populations into subpopulations with low, medium and high autophagy by flow cytometric cell sorting.

**Results:**

Olaparib and veliparib induced autophagy and cell death in parallel in WE-68 and SK-ES-1 cells. Induction of autophagy and cell death occurred at the same concentrations of both PARPi in both cell lines. Analysis of cells sorted according to autophagy levels revealed a clear association between PARPi effectiveness and autophagy level. The subpopulation of Ewing sarcoma cells with high autophagy responded significantly less to PARPi with cell death than the subpopulation with low autophagy.

**Conclusion:**

This study demonstrates that autophagy affects the anticancer activity of PARPi in Ewing sarcoma cells.

## Introduction

Ewing sarcoma (ES) is a highly aggressive cancer of bone and soft tissue, primarily affecting children, adolescents and young adults [1, 2]. Less than 10% of patients with ES survived before the introduction of chemotherapy. With the development of intensive multimodality therapy, consisting of multidrug cytotoxic chemotherapy along with surgery and/or radiotherapy, the prognosis improved steadily over the last decades of the twentieth century, leading to a five-year survival of around 70% overall in high-income countries [3]. However, the prognosis has improved only slightly since the turn of the century despite continuous attempts at treatment intensification [4]. This suggests that the chemotherapy protocols have been optimised to their limits and emphasises the need for new therapeutic approaches.

ES is caused by a pathognomonic chromosomal translocation in which an FET family gene fuses with an ETS family gene [1, 2]. In about 85% of cases, this translocation results in the EWSR1::FLI1 gene fusion encoding the chimeric oncoprotein EWS::FLI1 [5]. The latter is responsible for the initiation and progression of ES by acting mainly as a neomorphic transcription factor. Although EWS::FLI1 thus is a logical candidate target in ES, its direct targeting is challenging due to its function as a transcription factor [6, 7], and has had limited clinical success to date [8].

However, EWS::FLI1-mediated downstream aberrations may serve as druggable vulnerabilities [6, 7]. For example, EWS::FLI1 promotes R-loop accumulation and elevates replication stress, resulting in a ‘BRCAness’ phenotype [9]. ES is therefore particularly susceptible to agents that target proteins involved in replication stress and the DNA damage response [10–12]. Accordingly, inhibitors of DNA repair-associated proteins such as ATR, CDC7 or DNA-PK have demonstrated effectiveness against ES in preclinical studies [13–18].

The inhibition of poly(ADP-ribose)-polymerase (PARP) is considered to have particular potential in ES therapy [19, 20]. EWS::FLI1 drives PARP expression, which further increases EWS::FLI1-induced transcription in a positive feedback loop [21]. In line with this observation, ES cells were found to be as sensitive to PARP inhibitor (PARPi) treatment as BRCA1-deficient cancer cells, and EWS::FLI1 was identified as a biomarker for PARPi responsiveness [22]. However, although the PARPi olaparib was highly effective against ES in vitro [21–23], a phase II trial of olaparib in ES patients failed to deliver meaningful clinical benefits [24]. This indicates that PARPi monotherapy is not sufficient to achieve an objective response in ES [19].

Various approaches are being pursued to overcome the lack of response to PARPi in different cancer types [25–27]. In in vitro models of ES, PARPi cooperated with different cytotoxic agents to elicit cell death [28–30]. Phase I and II ES trials of PARPi in combination with either of the cytotoxic drugs irinotecan or temozolomide demonstrated limited antitumour activity [31, 32], while a phase I trial of the triplet combination with irinotecan and temozolomide suggested clinical benefit [33]. Yet haematological toxicity limited the clinical feasibility of dose escalation, implying that PARPi should rather be combined with non-cytotoxic agents [34]. A deeper understanding of the factors mediating PARPi resistance could therefore lead to superior combination partners that may benefit ES patients.

Autophagy is a fundamental cytoprotective activity [35] that has emerged as an important actionable resistance mechanism to chemotherapy and targeted therapy [36, 37], including PARPi [38]. PARPi have been found to induce autophagy, and cotreatment with autophagy inhibitors has been shown to enhance PARPi sensitivity in ovarian, hepatocellular, breast and prostate cancer cells [39–42]. Autophagy may also be a useful therapeutic target in ES, although its role is ambiguous, as it can function both as tumour promoter and suppressor [43]. The relevance of autophagy for the effectiveness of PARPi in ES, however, has not yet been examined. To explore this matter, we have used a flow-cytometric cell sorting procedure that separates ES cell populations into fractions with low, medium and high autophagy (hereafter referred to as Aut^LO^, Aut^ME^ and Aut^HI^, respectively). We found that the Aut^HI^ fraction was less sensitive to the PARPi olaparib and veliparib than the Aut^LO^ fraction, indicating that autophagy indeed is a resistance mechanism to PARPi in ES.

## Materials and methods

### Cell culture

WE-68 (RRID: CVCL_9717) cells were kindly provided by Dr F. van Valen (Münster, Germany), and SK-ES-1 (RRID: CVCL_0627) cells were purchased from the DSMZ (Braunschweig, Germany). Cells were cultured in RPMI 1640 medium (Capricorn Scientific, Ebsdorfergrund, Germany), supplemented with 10% foetal calf serum (Capricorn Scientific), 100 units/ml penicillin G sodium and 100 µg/ml streptomycin sulphate (Lonza, Basel, Switzerland). Cells were maintained throughout in rat-tail collagen-coated (5 µg/cm^2^; Merck, Darmstadt, Germany) cell culture vessels in a humidified atmosphere containing 5% CO_2_ at 37 °C and regularly passaged at a confluence of 90%. Mycoplasma contamination was routinely checked with the qPCR Mycoplasma Test Kit from Applichem (Darmstadt, Germany).

### Treatment of cells

Cells were seeded in 12-well tissue culture plates at a density of 150,000 cells/well. Twenty-four hours after seeding, cells were treated with the PARPi olaparib (0.1–4 µM) or veliparib (2–80 µM; Biomol, Hamburg, Germany) for 48 h. Olaparib and veliparib were dissolved in DMSO to prepare stock solutions and stored at − 80 °C. In the experiments shown in Fig. 2, cells were treated with PARPi for 24 h before treatment with the autophagy inhibitor chloroquine (CQ; 25 µM; Enzo Life Sciences, Lörrach, Germany) for 6–24 h. CQ was dissolved in PBS to prepare a stock solution and stored at 4 °C. In the cell sorting experiments, cells were seeded 2 h after sorting at a density of 100,000 cells/well and treated with PARPi for 48 h.

### Flow-cytometric analysis of cell death

Cell death was analysed by assessing the cell membrane integrity by flow-cytometric analysis of propidium iodide (PI; Sigma Aldrich) uptake. After harvesting, cells were incubated in 2 µg/ml PI in PBS at 4 °C immediately before analysis. 10,000 cells per sample were analysed on a BD (Heidelberg, Germany) FACSCanto II using BD FACSDiva software. Data were gated based on forward light scatter area (FSC-A) versus sideward light scatter area (SSC-A) to exclude debris and doublets.

### Flow-cytometric analysis of autophagy

Cells were stained with Cyto-ID (Enzo Life Sciences) according to the manufacturer’s recommendations. In brief, cells were washed with indicator-free medium (IFM), consisting of phenol red-free RPMI 1640 medium (Capricorn Scientific) containing 5% FCS and 2 mM L-glutamine (Lonza), and incubated in Cyto-ID at a dilution of 1:1000 in IFM for 30 min at 37 °C. After washing and resuspension in IFM, PI was added to a final concentration of 2 µg/ml immediately before analysis. 10,000 cells per sample were analysed on a FACSCanto II using FACSDiva software. Data were gated based on FSC-A versus SSC-A to exclude debris and doublets and further gated on PI-negative populations to exclude dead cells.

### Flow-cytometric analysis of mitochondrial transmembrane potential (Δ*ψ*_m_) dissipation

The loss of Δ*ψ*_m_ was analysed by determining 1,1’,3,3,3’,3’-hexamethylindodicarbocyanine iodide (DiIC_1_(5); Thermo Fisher Scientific, Dreieich, Germany) staining of mitochondria. Before harvesting, cells were incubated with 50 nM DiIC_1_(5) for 45 min at 37 °C in the dark. 10,000 cells per sample were analysed on a BD FACS Canto II using BD FACSDiva software. Data were gated based on FSC-A versus SSC-A to exclude debris and doublets.

### Flow-cytometric cell sorting based on Cyto-ID staining

The method for sorting ES cells based on Cyto-ID staining was adapted from our recently developed method for sorting leukaemia cells based on Cyto-ID staining [44]. Accordingly, 1 × 10^7^ cells were stained with Cyto-ID as described, resuspended in 1 ml IFM, filtered through 35-µm mesh and incubated with 1 µM Sytox Blue (Thermo Fisher Scientific) immediately before sorting. Cells were sorted into three subpopulations of approximately equal number according to the Cyto-ID fluorescence intensity, i.e., into Aut^LO^, Aut^ME^ and Aut^HI^ populations. To minimise the starvation period during the sorting procedure, the collection tubes were prefilled with 1 ml of complete growth medium or IFM, depending on the subsequent analysis. Sorting was done on a BD FACSAria Fusion at 45 psi using an 85 µM nozzle at 4 °C. Debris and aggregates were excluded from sorting using a sequential gating strategy relying on FSC-A versus SSC-A followed by FSC height (FSC-H) versus FSC width (FSC-W) and SSC height (SSC-H) versus SSC width (SSC-W) (see Fig. 3A). Dead cells were excluded by gating on Sytox Blue-negative cells.

### Statistical analysis

Statistical significance of differences between experimental groups was evaluated by a heteroscedastic, two-tailed Student’s *t*-test (corresponding to Welch’s t-test) using Microsoft Excel (*P < 0.05, **P < 0.01, ***P < 0.001).

## Results

### PARPi induce autophagy in parallel with cell death in ES cells

**Fig. 1:**
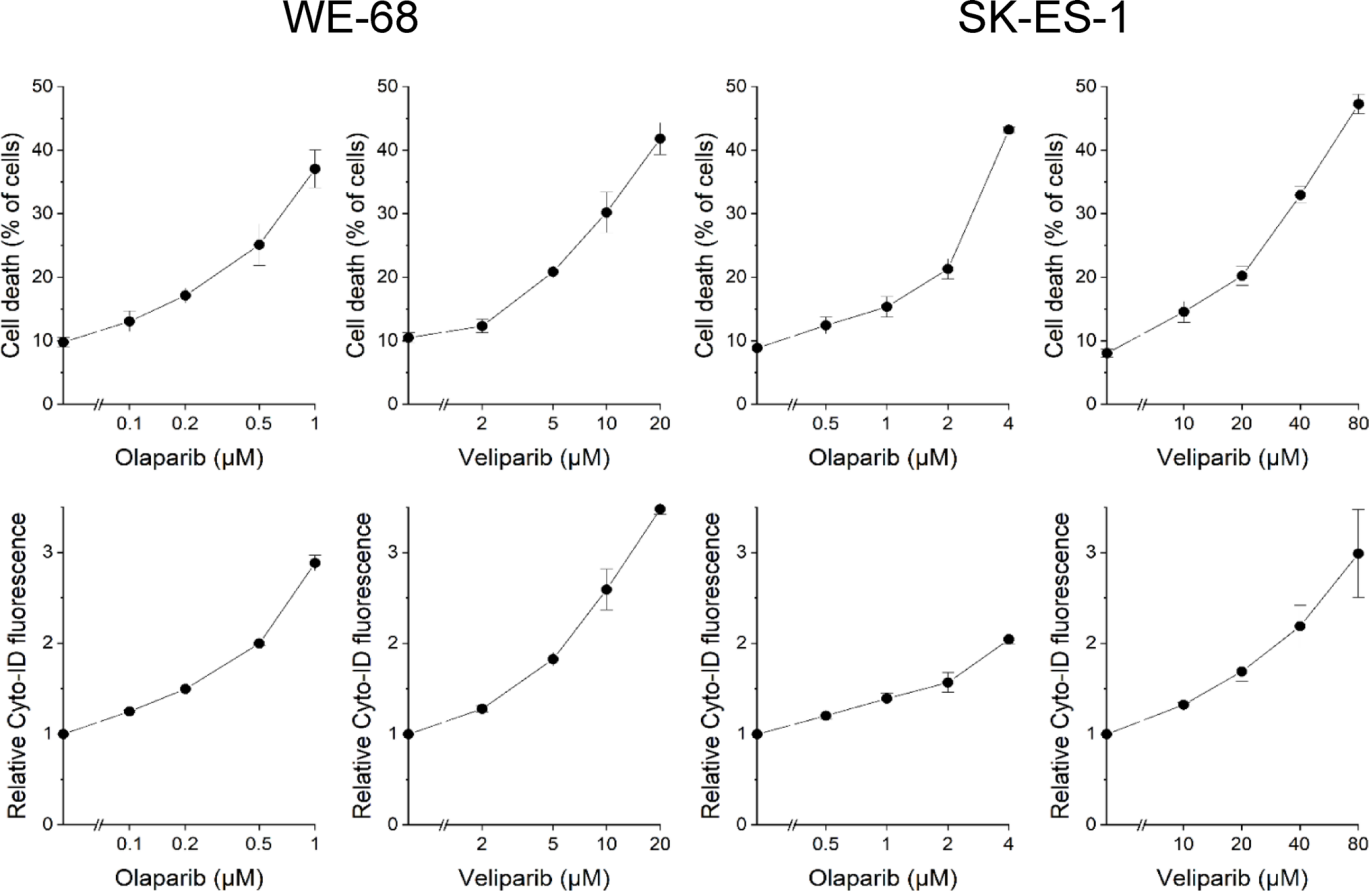
PARPi induce cell death and autophagy in ES cells. Cells were exposed to PARPi for 48 h. Cell death and autophagy were determined by flow-cytometric analysis of PI uptake and Cyto-ID staining, respectively. Cyto-ID fluorescence intensities were normalised to the mean Cyto-ID fluorescence intensities of untreated cells. Means ± SEM of three biologically independent measurements are shown

Since PARPi have been observed to induce autophagy in other tumours [39–41, 45], we initially tested whether PARPi would also induce autophagy in ES cells. To this end, we monitored the effect of two PARPi, olaparib and veliparib, on autophagy and cell death in two ES cell lines, WE-68 and SK-ES-1. Cells were exposed to PARPi for 48 h, and autophagy and cell death were assessed by flow cytometry. Autophagy was determined by Cyto-ID staining and cell death by PI uptake. Cyto-ID is a fluorescent dye that selectively labels autophagosomes [46–48], the key structures in the autophagic cascade [49]. Figure 1 shows that PARPi treatment induced cell death and autophagy in a concentration-dependent manner. Notably, the induction of cell death and autophagy followed the same pattern, with both drugs in both cell lines.

Linear regression analysis revealed a strong and statistically significant positive correlation between Cyto-ID fluorescence and PI uptake in both cell lines following PARP inhibition. This correlation was more pronounced in WE-68 cells (olaparib: R² = 0.966, *p* < 0.0001; veliparib: R² = 0.928, *p* < 0.0001) than in SK-ES-1 cells (olaparib: R² = 0.733, *p* < 0.001; veliparib: R² = 0.802, *p* < 0.001), suggesting a potentially cell line-dependent coupling between autophagy induction and cell death.

**Fig. 2:**
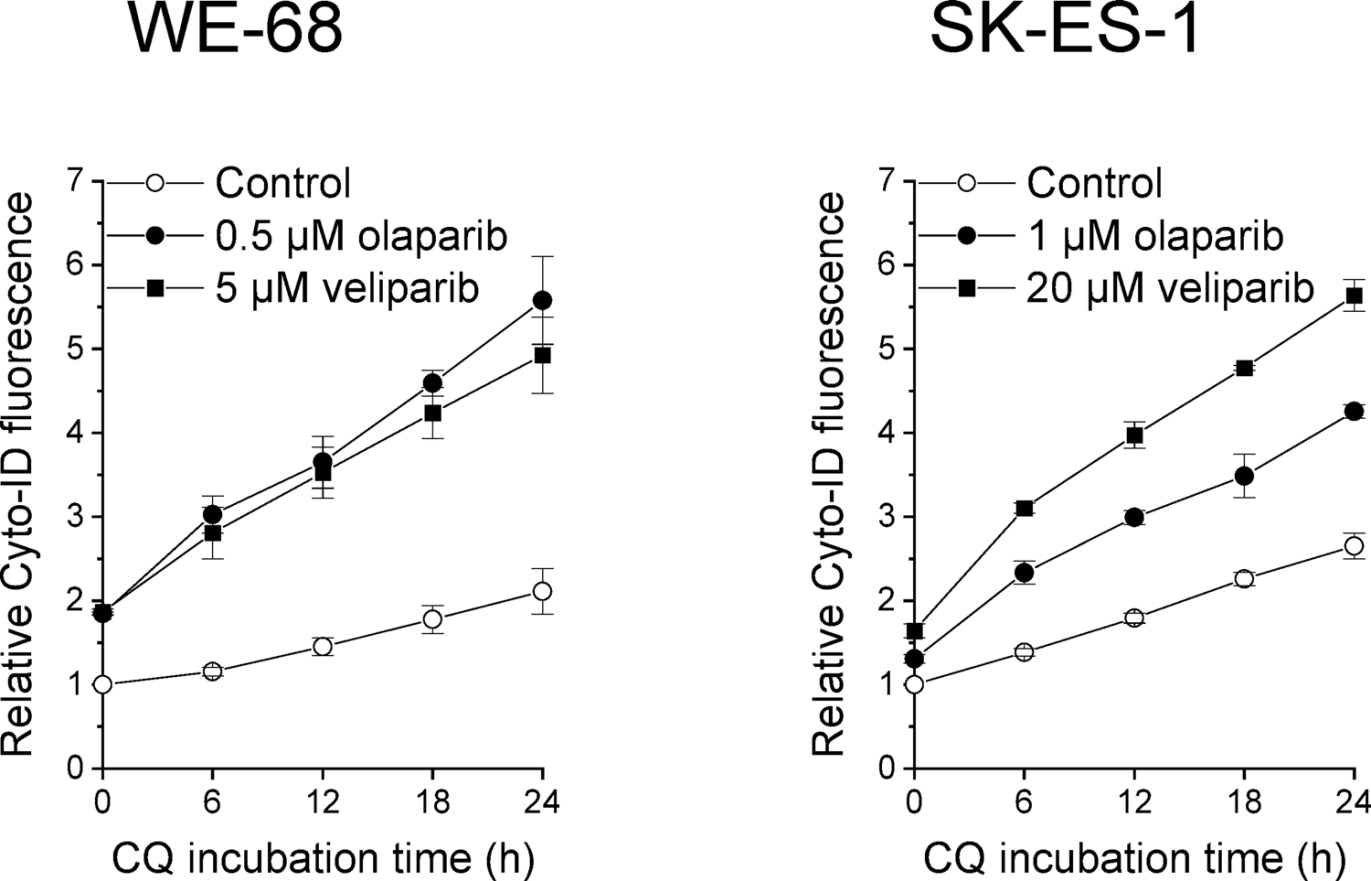
CQ enhances PARPi-induced increase in Cyto-ID fluorescence intensity in ES cells. After a 24-h treatment with PARPi, cells were exposed to 25 µM CQ for the indicated times. Autophagy was determined by flow-cytometric analysis of Cyto-ID staining. Cyto-ID fluorescence intensities were normalised to the mean Cyto-ID fluorescence intensities of untreated cells. Means ± SEM of two biologically independent measurements are shown

Similar to LC3 immunoblotting for the assessment of autophagy [50], Cyto-ID fluorescence alone is not sufficient to determine autophagic activity. However, the use of agents that block autophagosome turnover can provide evidence of changes in autophagy [50]. An additive or supra-additive effect of the combined treatment with the putative autophagy inducer and the autophagosome turnover blocker indicates enhanced autophagic activity. We therefore compared PARPi as single agents to PARPi in combination with the autophagosome turnover blocker CQ. The combination treatment resulted in a supra-additive effect compared to treatment with PARPi or CQ alone in WE-68 and SK-ES-1 cells (Fig. 2), evidencing that PARPi induced autophagy in ES cells.

### PARPi induce greater cell death in cells with low autophagy than in cells with high autophagy

**Fig. 3:**
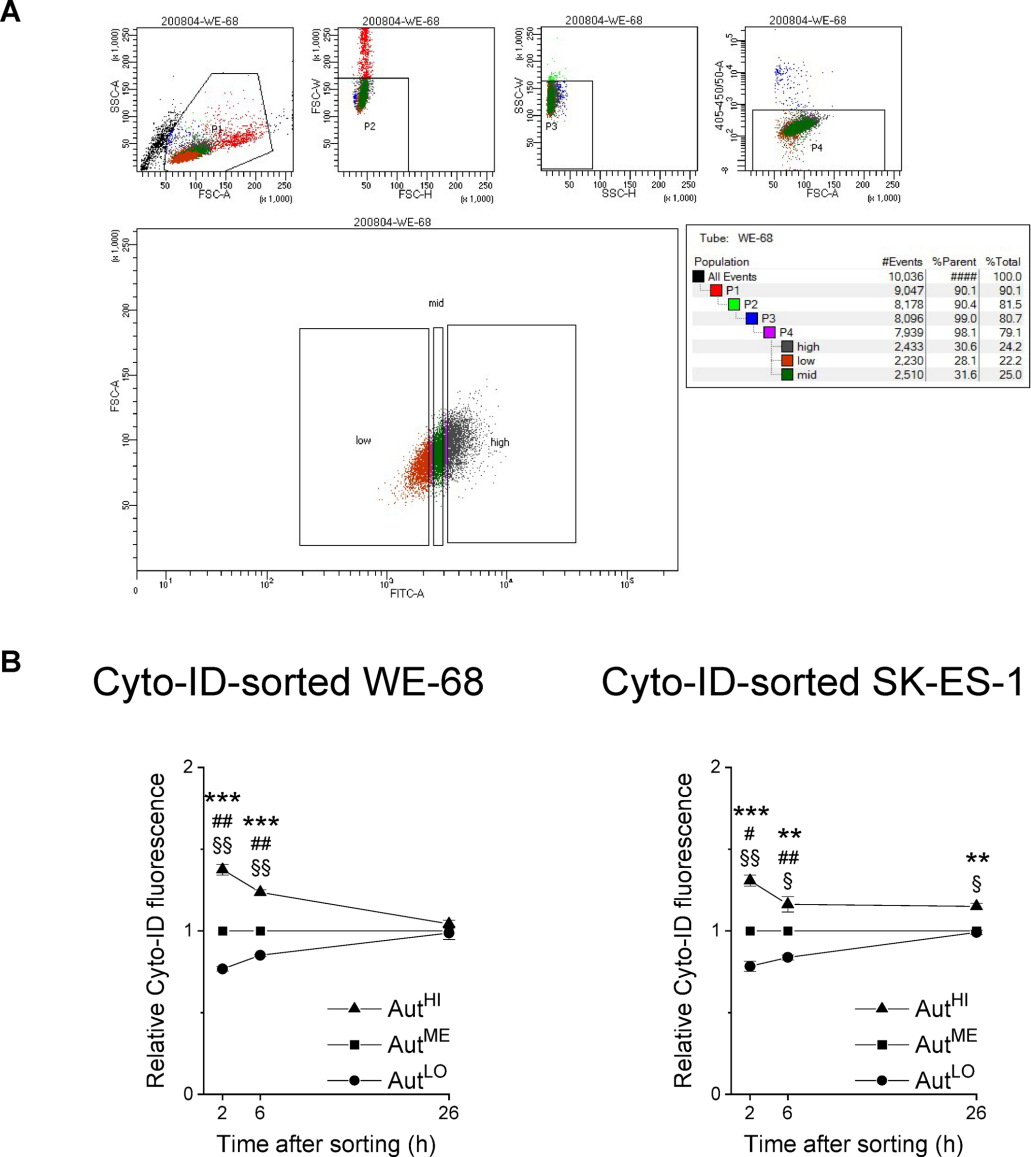
Cyto-ID-based sorting of ES cells. (**A**) Debris and aggregates were excluded from cell sorting using a sequential gating strategy relying on FSC-A versus SSC-A followed by FSC-H versus FSC-W and SSC-H versus SSC-W. Dead cells were excluded by gating on Sytox Blue-negative cells. Cells were sorted into three subpopulations of approximately equal number based on their Cyto-ID fluorescence intensities, i.e., into Aut^LO^, Aut^ME^ and Aut^HI^ subpopulations. (**B**) Cells were incubated for the indicated times after sorting, and autophagy was determined by flow-cytometric analysis of Cyto-ID staining. Cyto-ID fluorescence intensities of the three fractions were normalised to the Cyto-ID fluorescence intensity of Aut^ME^. Means ± SEM of three biologically independent measurements are shown (Aut^LO^ vs. Aut^HI^: *P < 0.05, **P < 0.01, ***P < 0.001; Aut^LO^ vs. Aut^ME^: ^#^*P* < 0.05, ^##^*P* < 0.01; Aut^ME^ vs. Aut^HI^: ^§^*P* < 0.05, ^§§^*P* < 0.01)

So far, our study has shown that PARPi induced autophagy in parallel with cell death in ES cells. To test whether autophagy indeed has an impact on PARPi-mediated cytotoxic effects, we utilised a recently developed Cyto-ID-based flow cytometric cell sorting method that separates cell populations into fractions with different basal autophagy levels, i.e., Aut^LO^, Aut^ME^ and Aut^HI^ cell fractions, suitable for downstream culturing of sorted cells [44]. Figure 3A depicts the gating strategy used for cell sorting based on Cyto-ID staining, the aim of which was to generate subpopulations of similar cell number with different autophagic activities. When determining the persistence of the Aut^LO^, Aut^ME^ and Aut^HI^ phenotypes, we observed that although the different autophagy levels tended to converge over time, they persisted for at least 6 h after sorting, and for 26 h in Aut^HI^ SK-ES-1 cells (Fig. 3B).

**Fig. 4:**
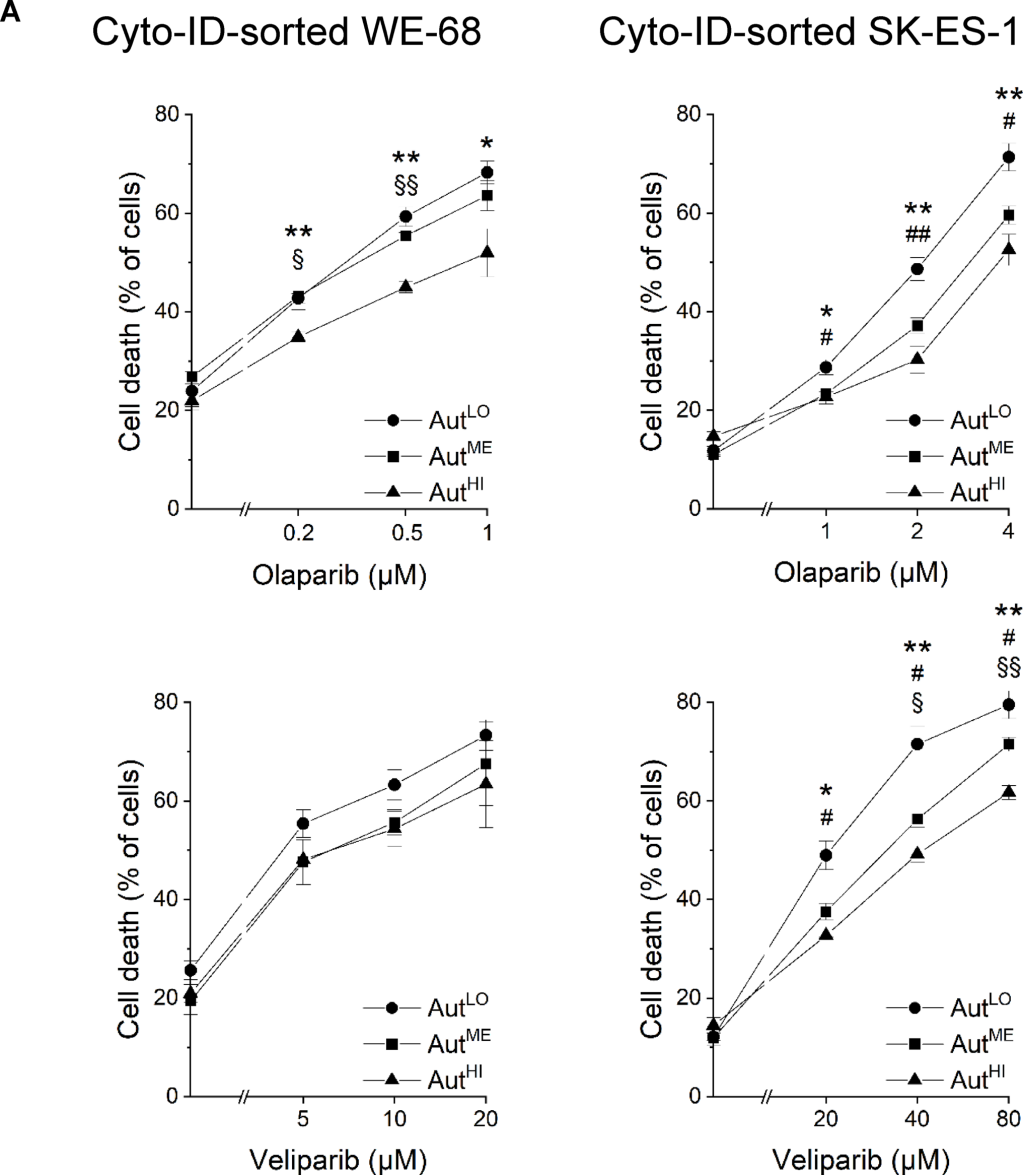

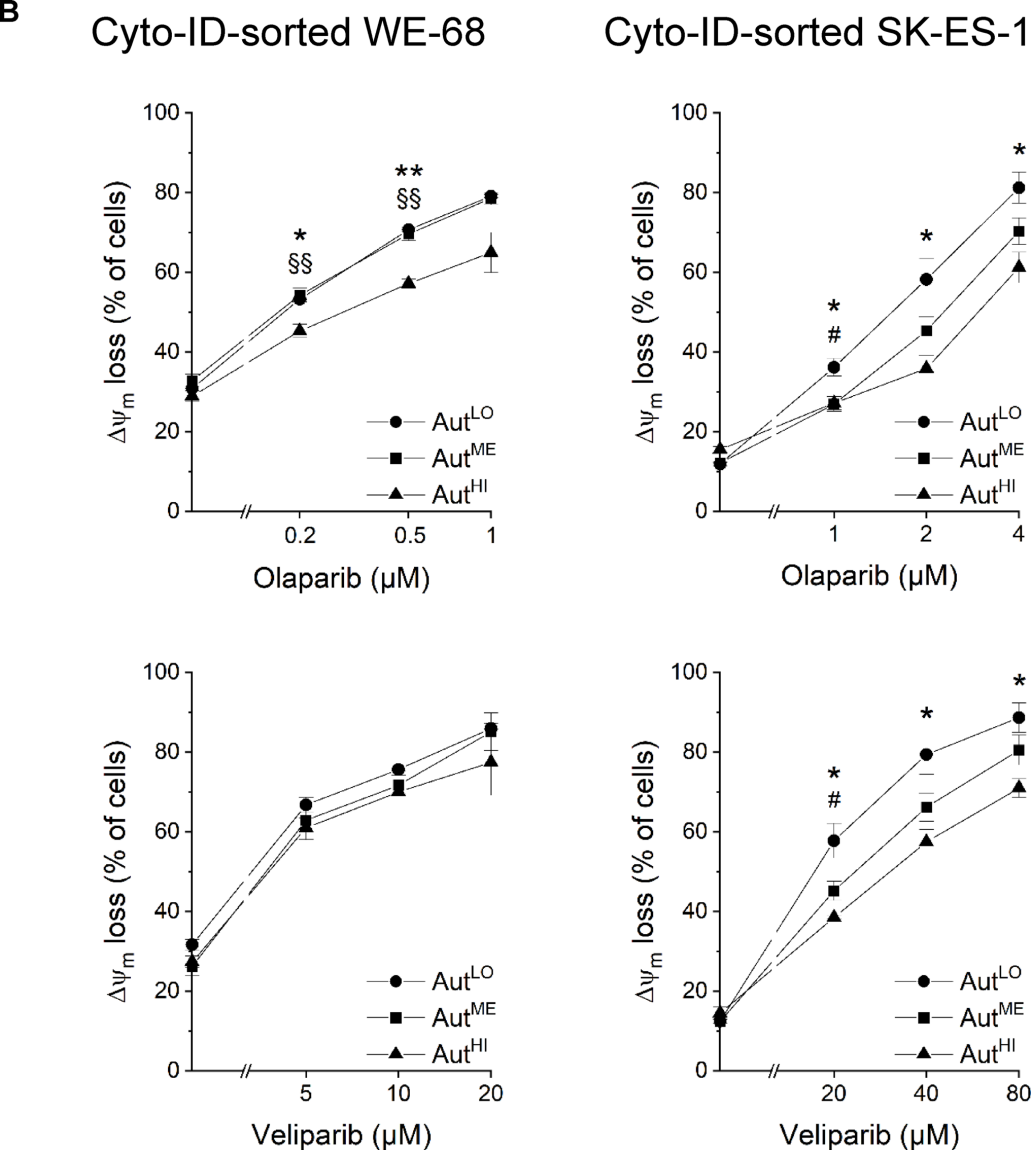
ES cells with high autophagy are less sensitive to PARPi than ES cells with low autophagy. Cells were flow-cytometrically sorted based on their Cyto-ID fluorescence intensity into Aut^LO^, Aut^ME^ and Aut^HI^ subpopulations. Two hours after sorting, cells were exposed to PARPi for 48 h. (**A**) Cell death and (**B**) loss of Δ*ψ*m were determined by flow-cytometric analysis of PI uptake and DiIC_1_(5) staining, respectively. Means ± SEM of three biologically independent measurements are shown (Aut^LO^ vs. Aut^HI^: *P < 0.05, **P < 0.01; Aut^LO^ vs. Aut^ME^: ^#^*P* < 0.05, ^##^*P* < 0.01; Aut^ME^ vs. Aut^HI^: ^§^*P* < 0.05, ^§§^*P* < 0.01)

To assess whether autophagic activity could affect the cytotoxic effectiveness of PARPi, we treated the Cyto-ID-sorted cell fractions with olaparib or veliparib and subjected them to flow-cytometric analysis of cell death and Δ*ψ*_m_ loss after 48 h. Figure 4A shows that the Aut^HI^ fractions of both cell lines showed significantly less cell death than the Aut^LO^ fractions upon olaparib treatment. For example, olaparib killed up to 68.3% of Aut^LO^ WE-68 cells, but only up to 52.0% of Aut^HI^ WE-68 cells. In SK-ES-1 cells, olaparib-induced cell death reached 71.4% in the Aut^LO^ fraction and 52.6% in the Aut^HI^ fraction. In the Aut^ME^ fractions, olaparib-elicited cell death was intermediate between that in the Aut^HI^ and Aut^LO^ fractions, even though the differences did not reach statistical significance in all cases. Veliparib induced significantly higher cell death in the Aut^LO^ fraction compared to the Aut^HI^ fraction in SK-ES-1 cells, but not in WE-68 cells.

Loss of Δ*ψ*_m_ served as a second read-out to monitor the cytotoxic effectiveness of PARPi in cells with different autophagy levels. Figure 4B shows that the effects of PARPi on Δ*ψ*_m_ loss in Cyto-ID-sorted WE-68 and SK-ES-1 cells were consistent with those on cell death. Again, the Aut^HI^ fractions of both cell lines responded significantly less with loss of Δ*ψ*_m_ upon olaparib treatment than the Aut^LO^ fractions. In the case of veliparib, a significant difference between the Aut^LO^ and Aut^HI^ fractions was again observed only in SK-ES-1 cells.

## Discussion

Preclinical studies indicate that PARPi offer a promising new therapeutic avenue for ES [19, 34, 51, 52]. However, translation of the preclinical findings to the clinic has not been crowned with success so far [24]. Therefore, the aim of current research is to identify the factors that determine PARPi resistance in ES to enable effective clinical use of this class of drugs [20]. Our study is the first to explore the impact of autophagy on the responsiveness of ES cells to PARPi.

In this study, we have found that PARPi promoted autophagy in parallel with cell death in ES cells. The induction of autophagy occurred at the same PARPi concentrations as the induction of cell death, suggesting that PARPi-mediated anticancer effects in ES cannot be achieved without concomitant activation of autophagy. It should be noted, however, that this does not necessarily imply a causal relationship between autophagy and cell death, but rather may suggest a pleiotropic effect of PARPi. In any case, PARPi may self-limit their anticancer effectiveness by inducing autophagy, as the latter plays a crucial role in the resistance of ES to antineoplastic drugs by acting as a survival mechanism under therapeutic stress [43]. To clarify whether autophagy is indeed a resistance mechanism to PARPi in ES, we compared the cytotoxic effectiveness of PARPi in ES cell populations with different autophagy levels. To this end, we applied a method we recently developed to sort leukaemia cells according to their autophagic activity [44]. Our previous study demonstrated the suitability of Cyto-ID for autophagy-based cell sorting.

The results obtained from separating ES cell populations into subpopulations with different autophagy levels revealed a clear association between PARPi effectiveness and autophagy level in ES cells: they responded to PARPi in the order Aut^LO^ > Aut^ME^ > Aut^HI^. It should in addition be taken into account that the differences in autophagy levels after sorting were transient, i.e., the autophagy in sorted cells persisted for at least 6 h after sorting but converged to baseline levels after 26 h in WE-68 cells. The results obtained may therefore even underestimate the actual effect of autophagy on PARPi effectiveness.

The transient nature of the differences in autophagy levels has another implication. EWS::FLI1 was shown to promote autophagy in ES cells by increasing the expression of ATG4B, a major regulator of the autophagic process [53]. Furthermore, a metabolism-associated gene signature, which includes autophagy-related genes, was developed for prognostic prediction in ES [54]. Similarly, the autophagy-related genes ATG2B, ATG10 and DAPK1 were found to have a potential protective function in ES [55]. It was thus concluded that the expression levels of autophagy-related genes may have prognostic value in ES. However, we observed a rapid reversion of the autophagy-sorted cell fractions, suggesting that the differences in autophagy levels were not due to genetic heterogeneity, but rather to stochastic fluctuations in autophagy activity in the initial cell population. A similar conclusion was reached in a study of autophagy-based sorted leukaemia cells, which showed that cell-to-cell differences in basal autophagy determined the apoptotic response to death ligands [56].

## Conclusion

Autophagy plays a complex role in ES and can have both tumour-promoting and -suppressive functions, depending on the context [43]. This ambiguous role of autophagy in ES thus advises against its immediate targeting without further investigation. In any case, however, the results presented here suggest that basal autophagy levels may affect the efficacy of PARPi in patients with ES – with higher levels of autophagy being associated with decreased susceptibility to PARPi.

## Data Availability

The datasets analysed during the study are available from the corresponding author on reasonable request.

## Abbreviations

Aut^HI^: High autophagy
Aut^LO^: Low autophagy
Aut^ME^: Medium autophagy
CQ: Chloroquine
DiIC_1_(5): 1,1’,3,3,3’,3’-hexamethylindodicarbocyanine iodide
ES: Ewing sarcoma
FSC-A: Forward light scatter area
FSC-H: Forward light scatter height
FSC-W: Forward light scatter width
IFM: Indicator-free medium
PARP: Poly(ADP-ribose)-polymerase
PARPi: PARP inhibitor
PI: Propidium iodide
SSC-A: Sideward light scatter area
SSC-H: Sideward light scatter height
SSC-W: Sideward light scatter width
